# Nc886 is epigenetically repressed in prostate cancer and acts as a tumor suppressor through the inhibition of cell growth

**DOI:** 10.1186/s12885-018-4049-7

**Published:** 2018-02-02

**Authors:** Rafael Sebastián Fort, Cecilia Mathó, Murilo Vieira Geraldo, María Carolina Ottati, Alex Shimura Yamashita, Kelly Cristina Saito, Katia Ramos Moreira Leite, Manuel Méndez, Noemí Maedo, Laura Méndez, Beatriz Garat, Edna Teruko Kimura, José Roberto Sotelo-Silveira, María Ana Duhagon

**Affiliations:** 1 Laboratorio de Interacciones Moleculares, Facultad de Ciencias, Universidad de la República, Montevideo, Uruguay; 2 Departamento de Genética, Facultad de Medicina, Universidad de la República, Montevideo, Uruguay; 3 Departamento de Biologia Celular e do Desenvolvimento, Instituto de Ciências Biomédicas, USP, São Paulo, Brazil; 4 Laboratório de Investigação Médica en Urologia, LIM55, Departamento de Urología, Faculdade de Medicina, USP, São Paulo, Brazil; 5 Departamento de Anatomía Patológica, Hospital Policial, Montevideo, Uruguay; 6 Departamento de Genómica, Instituto de Investigaciones Biológicas Clemente Estable, Montevideo, Uruguay; 7 Departamento de Biología Celular y Molecular, Facultad de Ciencias, Universidad de la República, Montevideo, Uruguay; 8 Present Address: Departamento de Diagnóstico y Tratamientos Especiales, Dirección Nacional de Sanidad de las Fuerzas Armadas, Hospital Central de las Fuerzas Armadas, Montevideo, Uruguay; 9 Present Address: Department of Structural and Functional Biology, Institute of Biology, Universidade Estadual de Campinas (UNICAMP), Campinas, Sao Paulo Brazil

**Keywords:** Cancer, Prostate, Metastasis, Vault RNA, nc886, vtrna2-1, miR-886, DNA methylation, Tumor suppressor, TCGA

## Abstract

**Background:**

Nc886 is a 102 bp non-coding RNA transcript initially classified as a microRNA precursor (Pre-miR-886), later as a divergent homologue of the vault RNAs (vtRNA 2–1) and more recently as a novel type of RNA (nc886). Although nc886/vtRNA2–1/Pre-miR-886 identity is still controversial, it was shown to be epigenetically controlled, presenting both tumor suppressor and oncogenic function in different cancers. Here, we study for the first time the role of nc886 in prostate cancer.

**Methods:**

Nc886 promoter methylation status and its correlation with patient clinical parameters or DNMTs levels were evaluated in TCGA and specific GEO prostate tissue datasets. Nc886 level was measured by RT-qPCR to compare normal/neoplastic prostate cells from radical prostatectomies and cell lines, and to assess nc886 response to demethylating agents. The effect of nc886 recovery in cell proliferation (in vitro and in vivo) and invasion (in vitro) was evaluated using lentiviral transduced DU145 and LNCaP cell lines. The association between the expression of nc886 and selected genes was analyzed in the TCGA-PRAD cohort.

**Results:**

Nc886 promoter methylation increases in tumor vs. normal prostate tissue, as well as in metastatic vs. normal prostate tissue. Additionally, nc886 promoter methylation correlates with prostate cancer clinical staging, including biochemical recurrence, Clinical T-value and Gleason score. Nc886 transcript is downregulated in tumor vs. normal tissue -in agreement with its promoter methylation status- and increases upon demethylating treatment. In functional studies, the overexpression of nc886 in the LNCaP and DU145 cell line leads to a decreased in vitro cell proliferation and invasion, as well as a reduced in vivo cell growth in NUDE-mice tumor xenografts. Finally, nc886 expression associates with the prostate cancer cell cycle progression gene signature in TCGA-PRAD.

**Conclusions:**

Our data suggest a tumor suppressor role for nc886 in the prostate, whose expression is epigenetically silenced in cancer leading to an increase in cell proliferation and invasion. Nc886 might hold clinical value in prostate cancer due to its association with clinical parameters and with a clinically validated gene signature.

**Electronic supplementary material:**

The online version of this article (10.1186/s12885-018-4049-7) contains supplementary material, which is available to authorized users.

## Background

Prostate cancer (PrCa) is the solid tumor with the highest incidence in men in Western countries, representing the second leading cause of male cancer death [1, 2]. Worldwide, one sixth of men will be diagnosed with prostate cancer in their lifetime. Although most patients can be treated successfully, a minor proportion develop an aggressive form of the disease that is currently incurable. It is fundamental to develop biomarkers that allow the precise prognosis at early stages, as well as new therapeutic tools to treat these patients in advanced stages. Non-coding RNAs have recently emerged as key players in cancer initiation and progression [3, 4], therefore their clinical value is under intense investigation [5–7].

The large collection of non-coding RNAs (ncRNAs) of the human genome is broadly grouped per size and function in two main types: a group of < 40 nt long RNAs known as “small RNAs” (including microRNAs, piwiRNAs, snoRNAs) and a group of > 200 nt long RNA named “long non-coding RNAs” [8]. The “vault” RNAs (vtRNAs) are a class of 84-141 nt long eukaryotic RNAs, that are transcribed by RNA polymerase III. They associate with conserved vault proteins forming the vault particle, a complex whose function and relevance in cancer remains scarcely understood [9]. Whereas the three human vtRNA1–1-3 are integral components of the vault particle, vtRNA 2–1 is a more divergent homologue, whose transcript is neither associated to the vault particle or co-regulated with the vtRNA1–1-3 [10, 11]. Before miRBase version 16, vtRNA2–1 was classified as a microRNA precursor, thus annotated as “precursor of hsa-miR-886-3p” (pre-miR-886); however, the recognition of its sequence homology with the three vtRNA-1 RNAs [11] led to its re-classification as vtRNA2–1 and the elimination of its derived microRNAs from miRBase. However, vtRNA2–1/pre-miR-886, was more recently proposed to be a new type of non-coding RNA (referred there as “nc886”), that acts as a tumor suppressor, inhibiting the activation of Protein Kinase RNA-activated (PKR) by direct interaction [12–14]. Consistent with these findings, Treppendahl et al. showed that nc886 functions as an epigenetically regulated tumor suppressor gene in acute myeloid leukemia, and that genome demethylating treatment inhibits PKR phosphorylation [15]. However, in the same work, the authors detected mature miRNAs derived from nc886, and showed they are products of the processing of pre-miR-886 by a non-canonical pathway independent of DROSHA. In addition, other groups have identified the mature microRNAs derived from pre-miR-886 in lung small cell carcinoma [16] and prostate cancer [17], presenting evidence of its association with disease progression.

Different lines of evidence have revealed that the epigenetic control of nc886 is complex and may own clinical relevance. Independent reports in breast, lung, colon, bladder, esophagus and stomach cancer showed that its promoter is differentially methylated in tumor vs. normal tissue [18, 19]. In fact, in lung cancer, chronic myeloid leukemia and gastric cancer, its differential methylation correlates with patient prognosis and survival [15, 16, 20]. Although these findings support a tumor suppressor role for nc886, a recent communication proposed its action as an oncogene in thyroid cancer [21]. Intriguing aspects of the epigenetic regulation of this locus, include its dependence on the parental origin of the allele [22], and its sensitivity to the peri-conceptional environment [23].

The aim of this study was to investigate the possible involvement of nc886 in PrCa etiology and behavior. Analyzing clinical samples, we found that the full transcript of nc886 is present in prostate tissue and diminishes its abundance in tumor compared to normal tissue, thus showing a gene expression pattern of a tumor suppressor gene. The increased methylation of nc886 promoter in transformed vs. non-transformed tissue, as well as demethylating agent treated vs. untreated cell lines, indicate that the molecular etiology of nc886 downregulation is the methylation of its promoter. Indeed, nc886 promoter methylation level correlates with clinical parameters of PrCa (Gleason Score, clinical T value and biochemical relapse). Forced restitution of nc886 in DU145 and LNCaP cell lines produces an inhibition of cell invasion and proliferation in vitro and a reduction of DU145 tumor growth in vivo. These results are consistent with a tumor suppressor role, suggesting a nc886 antiproliferative function in normal prostate tissue. Finally, the interrogation of the Prostate Adenocarcinoma of The Cancer Genome Atlas (TCGA-PRAD) cohort, uncovered a negative association between the expression of nc886 and the expression of genes belonging to the PrCa cell cycle progression gene signature (CCP), providing a molecular support for the phenotype experimentally observed after nc886 recovery.

## Methods

### Human specimens

Tissue sections were obtained from paraffin fixed blocks stained with hematoxylin and eosin (H&E) of 6 archived radical prostatectomies and were evaluated by three pathologists at the Department of Anatomic-pathology of the Police Hospital. This study was approved by the Hospital Policial, D.N.AA.SS., Montevideo, Uruguay (2010).

Matched normal and tumor regions, showing similar parenchyma-stroma ratio and similar cytological findings at the stroma were selected. Unstained section of 10-μm thickness, contiguous to the sections selected by the pathologist, were then freshly obtained to extract small RNAs using the RNeasy FFPE (Qiagen) Kit, with the following modifications: two extra washes with xylene and absolute ethanol were added. The RNA was resuspended in RNAse free water and stored at − 20 °C for further analysis.

### Cell lines

RWPE-1, LNCaP (ATCC CRL-1740), PC-3 and DU145 human prostate cancer cell lines were obtained from ATCC (Manassas, VA, USA). LNCaP, DU145 and PC-3 were maintained in RPMI 1640 (R7755) supplemented with 10% FBS (PAA™) and penicillin/streptomycin. RWPE-1 cell line was cultured in Keratinocyte Serum Free Medium (Gibco by LifeTechnologies™) supplemented with 0.03 mg/mL bovine pituitary extract (BPE) and 0.5 ng/mL EGF human recombinant epidermal growth factor (EGF) and penicillin/streptomycin. All cell lines were maintained in a 5% carbon dioxide atmosphere at 37 °C.

A lentiviral vector bearing the precursor nc886 or a scrambled sequence of the same length, both cloned downstream of the CMV promoter (miExpress precursor expression clones, pEZX-MR02, GeneCopoeia) were transduced in DU145 and LNCaP. Transduced cells were then selected by growth in the presence of puromycin.

### 5-Azacytidine treatment

DU145, RWPE-1, PC-3 and LNCaP cells were treated for 72 h with 1.5 μmol/L 5-Azacytidine (ab142744, Abcam) and DMSO as control, replacing the medium with freshly added drug every 24 h following manufacturer’s instructions.

### RNA extraction, reverse transcription and quantitative real time PCR

Total RNA was extracted using the Qiagen™ miRNAeasy kit. Reverse transcription was performed using the Qiagen PCR miScript II System. Quantitative real time PCR (qPCR) was performed with the miScript SYBR Green PCR Kit using specific oligonucleotides. For nc886: 5′CGGGTCGGAGTTAGCTCAAGCGG3′ forward primer and 5′AAGGGTCAGTAAGCACCCGCG3′ reverse primer, as in Lee K et al. [12]. U6 RNA was amplified using the primer assay purchased from Qiagen (Hs_RNU6-2_11 miScript Primer Assay (MS00033740)) and the miScript Universal Primer (Qiagen). The relative quantification was attained using the ΔΔCT method, in a Rotor-Gene 6000 equipment (Corbett Life Science), employing U6 as the internal control of RNA load.

### MTT assay

Five thousand DU145 and LNCaP cells per well were seeded in 96 culture plates. Twenty μL of 3-(4,5-dimethylthiazol-2-yl)-2,5-diphenyl-2H-tetrazolium bromide (MTT) 5 mg/mL solution dissolved in 1X PBS was added to the wells and cultures were incubated for 4 h at 37 °C in a 5% CO_2_ controlled atmosphere. The medium was then aspirated and 100 μL of DMSO was added to each well and incubated at room temperature in the dark for 15 min with moderate orbital shaking. Optical density (OD) was read in a plate spectrophotometer (Thermo Scientific Varioskan® Flash Multimode) at 570 nm and 690 nm wavelengths.

### Flow cytometry for DNA content

DU145 cells transfected with lentiviral vectors producing nc886 or a scrambled RNA control were seeded in triplicate in 6-well plates. Upon reaching 60% confluence, cells were harvested by trypsinization, washed twice with 1X PBS and resuspended in 1X PBS by gentle vortexing. Cells were then fixed by adding 1 mL of ice cold 70% ethanol dropwise in 1X PBS and incubated at − 20 °C for 30 min. Next, cells were washed with 1X PBS and centrifugated at 1200 rpm at 4 °C for 5 min and the resuspended cell pellets were incubated with 0.1 mg/mL of RNase and 50 μg/mL propidium iodide for 15 min at room temperature in the dark. Flow cytometry measurement of nuclear DNA content was performed in a Accuri™ C6 flow cytometer (BD Bioscience), counting 10.000 total events per sample (BD Accuri C6 software).

### Matrigel invasion assays

24-well transwell inserts of 8 μM pore size (Corning #3422) were coated with Matrigel (Corning) for in vitro invasion assays. Fifteen thousand (DU145) or 20,000 (LNCaP) cells were seeded in serum-free RPMI 1640 and migrated towards the bottom chamber containing RPMI 1640 supplemented with 10% FBS. After 48 h the cells were fixed with 100% methanol and stained with hematoxylin and eosin (H&E). Non-invading cells were scrubbed with a cotton swab. Five microscopic fields were photographed and counted for each sample. Values were averaged from at least 3 independent experiments.

### Mice xenograft

Six 4-week-old male athymic NUDE BALB/C mice were maintained according to the protocols and ethical regulations of the animal facility of the Institute of Biomedical Science, at the University of Sao Paulo, Brazil (protocol 134/10, approved by the Ethics Committee for Animal Use). In order to grow tumors, these mice were subcutaneously injected on both flanks using 3 × 10^6^ DU145 cells resuspended in 50 μl of Matrigel matrix (Corning Inc.) per inoculation. The tumor growth was measured weekly with calipers and the corresponding volumes were calculated as: length x width x height x π/2. When tumors reached 2 cm, the animals were euthanized, and tumors were extracted and properly stored for further analysis.

### Analysis of mice tumors

The study of histological sections of the tumors extracted from the mouse xenotransplantation assays, was conducted at the Laboratory of Medical Research – LIM55, Urology Department, of the University of Sao Paulo, Brazil. Specifically, the percentage of necrosis and mitotic indexes in the histological sections of the tumors stained with H&E were quantified.

### Dataset analysis

The TCGA-PRAD data was downloaded from the TCGA portal (https://tcga-data.nci.nih.gov/docs/publications/tcga/?) [24] and the Methhc database (http://methhc.mbc.nctu.edu.tw/php/index.php) [25]. This dataset includes the RNA-Seq expression values of 50 matched normal and tumor tissue and additional unmatched normal and tumor samples, generated using Ilumina sequencing technology. The methylation data of the TCGA-PRAD cohort, was extracted from the Illumina Infinium Human Methylation 450 BeadChip array data of the 49-paired normal and prostate tumor samples and additionally unmatched normal and tumor tissues (336 in total). Several public methylomes available at the Gene Expression Omnibus (GEO) repository [26] were also analyzed: matched normal and tissue PrCa GSE76938 [27], PrCa metastasis GSE38240 [28], PrCa cell lines GSE34340, GSE62053 and GSE54758 [29, 30] and HCT166 cell lines GSE51810 [31]. The average of the normalized beta-values for the 6 CpGs sites located at the nc886 TSS200 promoter (cg18678645, cg06536614, cg26328633, cg25340688, cg26896946, cg00124993) were calculated. Hierarchical clusterization obtained through Euclidean algorithm was performed using the Gene-E (http://www.broadinstitute.org/cancer/software/GENE-E/) for the methylation beta-values and Morpheus (https://software.broadinstitute.org/morpheus/) for gene expression values.

### Statistical analysis

All experiments were performed at least in triplicate, and the corresponding variables are expressed as average value ± standard deviation or standard error. Statistical analyses were done using single and two-tailed t-test, and the statistical significance of the observed differences were expressed using the *p*-value (* *p* < 0.05, ** *p* < 0.01, *** *p* < 0.001). D’Agostino-Pearson was conducted as the normality test and nonparametric Spearman was used to test correlation.

## Results

### Nc886 promoter methylation is increased in neoplasic relative to normal prostatic tissue and correlates with biochemical recurrence and tumor grade

**Fig. 1:**
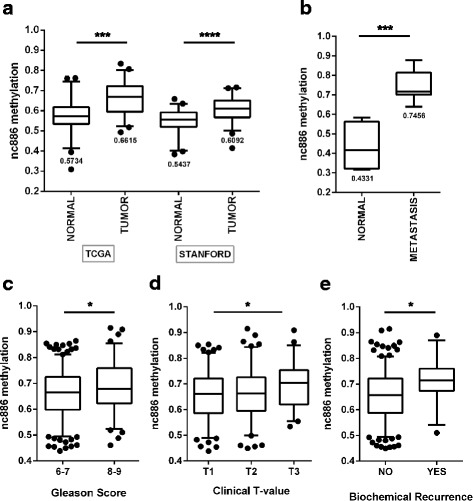
Nc886 TSS200 methylation status and its correlation with clinical parameters of PrCa in patients of the TCGA-PRAD cohort. **a** Average TSS200 methylation of nc886 in matched normal and tumor prostate tissue of 50 patients from TCGA cohort and 52 patients from STANFORD cohort data available in GSE76938 GEO dataset. **b** Average TSS200 methylation of nc886 for normal and metastatic prostate tissue data available in GSE38240 GEO dataset. In A and B the numbers below the boxes indicate the mean value of each distribution. **c** Average TSS200 methylation of nc886 in association with Gleason Score of tumor tissue of 329 patients. **d** Association between average TSS200 methylation of nc886 and clinical T values of tumor tissue of 272 patients. **e** Association between average TSS200 methylation of nc886 and biochemical recurrence in 276 patients. Clinical data analyses incorporate TCGA-PRAD DNA methylation data of the tissues with available associated Gleason score data (**c**), Clinical T-value data (**d**) and Biochemical recurrence data (**e**). Two-tailed T test was performed for all categories, and only significant differences are depicted: * *P* value < 0.05; ** *P*-value < 0.01; *** *P* -value < 0.001; **** *P*-value < 0.0001

In view of the existing background about the role of nc886 promoter methylation in other types of cancer [15, 16, 18, 19], we analyzed the methylation levels of its proximal promoter in PrCa. For that, we selected the region of 200 nt located upstream of the transcription start site (TSS200) of nc886 in the genome wide methylation microarray data available at TCGA-PRAD. The analysis of the available 50 paired tissue samples showed a statistically significant increase of the methylation average in tumor (0.6615 ± 0.08215) relative to normal tissue (0.5734 ± 0.08049) (*p*-value < 0.001) (Fig. 1a). An identical analysis was performed using a recently published PrCa cohort [27] comprising 52 matched cancer and benign-adjacent tissue of radical prostatectomies from Stanford University Medical Center, yielding very similar results (0.5437 ± 0.06445 normal vs 0.6092 ± 0.06278 tumor) (Fig. 1a). It is worth to note that nc886 TSS200 methylation is quite variable among the samples. Nevertheless, regardless of the initial methylation status, the tumor tissue consistently shows a higher methylation relative to the adjacent normal tissue, as depicted in the TSS200 methylation clustering of patient samples presented in Additional file 1: Figure S1.

Seeking to investigate the relevance of nc886 promoter methylation in the metastatic stage of PrCa, we analyzed its status in 4 normal prostates from organ donors and 8 PrCa metastases in a rapid autopsy cohort of lethal metastatic PrCa available at the GEO [28]. We observed that the metastases have a significant higher average nc886 promoter methylation (0.7456 ± 0.0771) than the normal tissue (0.4331 ± 0.1283) (*p*-value < 0.001) (Fig. 1b) of the same study. Interestingly, the metastatic tumors present similar levels of nc886 promoter methylation (0.7456 ± 0.0771) than the highest methylated group of samples among the primary tumors (0.7239 ± 0.0478) and normal tissues (0.6945 ± 0.0386) defined in Additional file 2: Figure S2. To assess the clinical relevance of nc886 promoter methylation, we studied its association with the clinical data of the patients of the TCGA- PRAD cohort. We found that nc886 TSS200 average methylation is significantly associated with Gleason score (p-value < 0.05), clinical T-value (p-value < 0.05) and biochemical relapse (p-value < 0.01) (Fig. 1c-e). We also found that the nc886 methylation status in the normal prostatic tissue dissected from prostatectomies [19], calculated with the 10 selected CpG sites (Additional file 2: Figure S2), is associated with the clinical T-value of the matched patient tissue (Additional file 2: Figure S2D) (p-value < 0.05).

### Increased promoter methylation of nc886 causes a reduction of the transcript level in prostate cancer

**Fig. 2:**
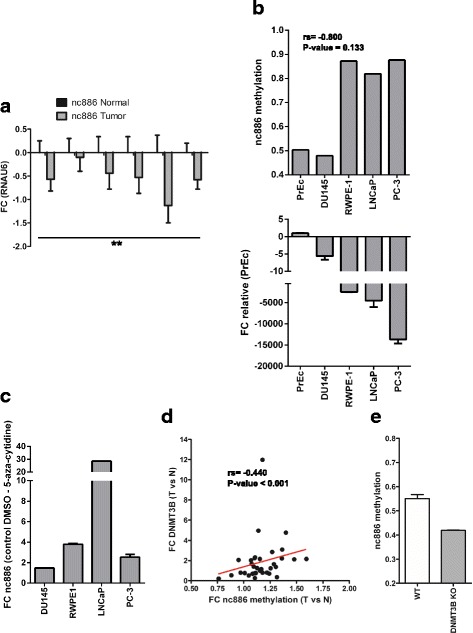
Nc886 expression and its correlation with promoter methylation in prostate cells. Expression levels of nc886 were determined using RT and qPCR with specific primers (see Materials and Methods) and RNAU6 was used as and endogenous control of RNA amount (A, B and C). **a** Expression of nc886 in 6 matched normal and tumor human prostate tissue relative to RNAU6. **b** Expression of nc886 relative to PrEC are presented (upper plot) and average methylation of nc886 promoter (lower plot) in prostate cancer cell lines. Methylation data was extracted from publicly available GEO datasets GSE34340, GSE62053 and GSE54758. **c** Expression of nc886 in prostate cancer cell lines treated with 5-Azacytidine relative to untreated cell lines (control DMSO). **d** Correlation between the fold change in average nc886 TSS200 methylation and DNMT3B expression in tumor vs. normal tissue, assessed in 50 matched samples of the TCGA-PRAD dataset. **e** Average methylation of nc886 promoter 10 CpG sites of wt HCT116 and DNMT3B KO HCT116 cell line (GEO dataset GSE51810). P-value < 0.05 t-test two-tailed. * P-value < 0.05; ** P-value < 0.01; *** P-value < 0.001; two tailed t-test. The correlation was conducted by D’Agostino-Pearson normality and nonparametric Spearman tests

Although growing evidence is demonstrating a tumor suppressor role for nc886 in several types of cancer, there is fewer assessment of the molecular etiology of its downregulation by promoter methylation during carcinogenesis. Thus, we sought to find a direct association between nc886 transcripts levels and the methylation of its promoter in PrCa clinical samples. We first investigated the presence and deregulation of the full nc886 transcript in PrCa. Consequently, we isolated RNA from six paired normal and tumor samples from paraffin blocks obtained from radical prostatectomies, and we performed RT-qPCR with oligonucleotides specific for the nc886 transcript. We found that nc886 transcript is significantly suppressed in tumor relative to normal tissue, with an average fold change in expression of − 0.56 ± 0.14 (Fig. 2a). Furthermore, the interrogation of several datasets available at GEO showed that the average methylation value of nc886 promoter in prostate cell lines (PrEC, DU145, RPWE-1, LNCaP and PC-3) inversely correlates with the levels of its transcript (rs = − 0.80) (Fig. 2b). In addition, the treatment of DU145, LNCaP, RWPE-1 and PC-3 cell lines with the DNA demethylating agent 5-Azacytidine increases nc886 levels (Fig. 2c). As seen in the clinical specimens, the level of nc886 expression/nc886 methylation in the PrCa cell lines is variable (Fig. 2b). Although we know the nc886 status in the matched normal/tumor tissue of the clinical specimens (Fig. 1a), we unfortunately do not know the nc886 status in the normal prostate cells of the cell line donor patients. However, based on the clinical data, nc886 expression is expected to be higher in the non-transformed cells of the donor. In this context, what is relevant for the hypothesis is the decrease in nc886 expression during malignant transformation independently of the initial status of the gene. Interestingly, nc886 status separates the cell lines in two groups, one comprising PrEc and DU145 and another comprising RWPE-1, LNCaP and PC3. These two groups of cell lines are representative of the variable nc886 status in the prostate tissues. Therefore, DU145 and LNCaP/PC-3 PrCa cell lines represent suitable models for the spectrum of nc886 variability observed in the clinical set.

Patterns of DNA methylation during development and carcinogenesis are established by DNA methyl transferases, comprising maintenance DNMT1 and de novo DNMT3A and DNMT3B. To investigate which of these enzymes would be responsible for the increase in nc886 TSS200 methylation in prostate carcinogenesis, we compared the expression of the DNMTs with the level of nc886 TSS200 methylation in TCGA-PRAD tumor samples. Of the 3 DNMTs both DNMT3A and DNMT3B increase their transcript level in tumor compared to matched normal samples of this cohort (Additional file 3: Table S1), as calculated from the RNA-seq data. Interestingly, nc886 TSS200 average methylation positively correlates with the expression of DNMT3B, and DNMT3A, but not DNMT1 (Additional file 3: Table S1). More interestingly, a correlation between the fold change of expression of DNMT3B and the fold change in nc886 methylation in the 34-paired normal vs. tumor tissues is observed (rs = 0.4402, *p*-value < 0.001, Fig. 2d and Additional file 3: Table S1). Further support for the specific role of DNMT3B in nc886 promoter methylation is provided by the analysis of a GEO dataset [31], which uncovered a drastic reduction in its methylation upon the deletion of DNMT3B in the HCT116 cell line (Fig. 2e).

### Overexpression of nc886 causes a decrease in tumor growth in vitro and in vivo

**Fig. 3:**
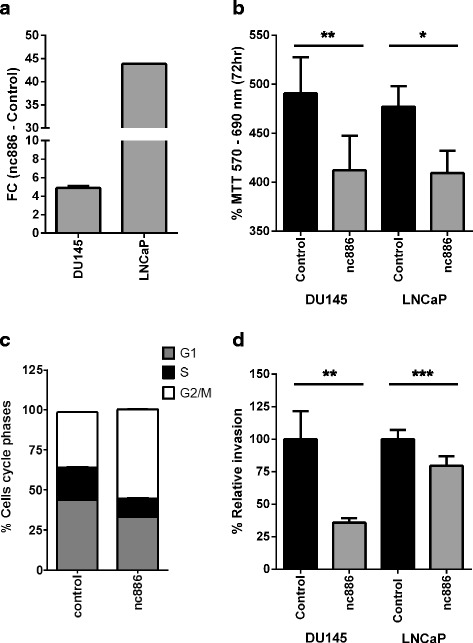
In vitro effect of the overexpression of nc886 in tumor cell proliferation and invasion. **a** Expression of nc886 in DU145 and LNCaP nc886 overexpressing relative to control cell line. Expression levels of nc886 were determined using RT and qPCR with specific primers (see Methods) and RNAU6 was used as and endogenous control of RNA amount. **b** Cell viability assay by MTT for DU145 and LNCaP cell lines overexpressing nc886 and control hairpin RNA. The absorbance at 72hs (570-690 nm) is shown as percentage. **c** Flow cytometric cell cycle assay based on DNA content measured with propidium iodide. The cumulative percentage of cells at the different cell cycle phases are shown for DU145 cell line overexpressing nc886 and control hairpin RNA. **d** Matrigel invasion assay of DU145 (3 replicates) and LNCaP (4 replicates) overexpressing nc886 and control hairpin RNA. Percentage invasion for nc886 overexpressing cell lines was calculated relative to control cell lines. * P value < 0.05; ** P-value < 0.01; *** P-value < 0.0001 two tailed t-test

Since the pattern of expression and promoter methylation of nc886 in PrCa suggested that nc886 could function as tumor suppressor gene in the prostate, we decided to investigate the phenotypic consequence of nc886 transcript recovery. The overexpression of nc886 was forced into the cell line DU145 and LNCaP by stable transfection of a lentiviral vector encoding nc886 under CMV promoter regulation. A control vector, overexpressing a random RNA sequence, which has no complementarity with human genomic sequences, was used as a control in all the experiments. The overexpression of nc886 was confirmed by RT-qPCR, demonstrating an increase of nc886 of 4.9 ± 0.2 in DU145 and 43.87 ± 0.01 in LNCaP (Fig. 3a). The fold change in expression in DU145 transfectant mimics the difference observed between the expression of nc886 in the malignant DU145 vs. the normal PrEC cell line (Fig. 2b); it is also comparable with the fold change in nc886 expression observed in the tumor vs. normal tissue of the clinical samples (Fig. 2a). Since one of the essential hallmarks of malignant transformation is the increased cell proliferation, we evaluated this phenotype using the cell viability MTT assay. We found that DU145 and LNCaP cell lines overexpressing nc886 have a lower rate of proliferation relative to the corresponding control cell lines (Fig. 3b). To study this phenotype in greater depth, we performed an analysis of DNA content by flow cytometry in DU145, whose results revealed an enrichment of cells in G2/M phase in the nc886 overexpressing transfectant compared to the control (Fig. 3c).

**Fig. 4:**
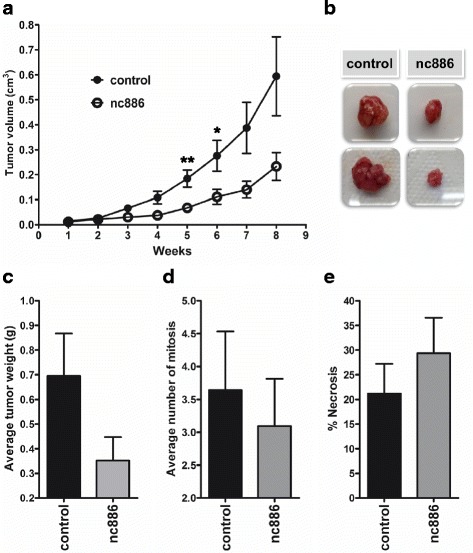
In vivo effect of nc886 overexpression in tumor phenotype. **a** Tumor xenograft assay in NUDE BALB-C mice of DU145 cell lines overexpressing nc886 and control hairpin RNA. Tumor growth curves of 6 mice expressed as the average tumor volume (cm^3^). **b** Macroscopic images of selected tumors grown in NUDE BALB-C mice inoculated in with DU145 cell lines overexpressing nc886 and corresponding control cell line. The average mass(g) (**c**), average number of mitosis (**d**) and average percentage of tumor necrosis (**e**) of the 6 tumors assayed at the end time of the assay are shown. * *P* value < 0.05; ** *P*-value < 0.001; two-tailed t-test

To investigate whether this anti-proliferative effect also took place in vivo, we performed a xenograft assay in NUDE BALB-C mice. Specifically, 6 male mice were subcutaneously inoculated in opposite flanks with DU145 cell line overexpressing nc886 or the control vector. As shown in Fig. 4a and b, the tumors resulting from the DU145 control cell line had a significantly higher growth relative to the tumors of the DU145 cell line overexpressing nc886. This growth difference is also reflected in the mass of the tumors (Fig. 4b and c). Additionally, the histology of the tumors was analyzed by optical microscopy of paraffin derived tumor sections stained with hematoxylin and eosin. This showed a trend towards a higher mitotic index and a lower percentage of necrosis in tumors of the control cell line compared to the tumors overexpressing nc886 (Fig. 4d and e). Thus, the results obtained in vivo reinforce those previously observed in vitro.

### Overexpression of nc886 causes a decrease in tumor cell invasion in vitro

The ability of the tumor cells to cross the extracellular matrix (which in epithelia is represented by the basement membrane) and invade surrounding and distant tissues is a fundamental hallmark of malignancy. Taking in consideration the increment in nc886 promoter methylation in metastatic relative to normal tissue observed in the cohort of Aryee et al. (Fig. 1b), we sought to investigate the effect of its overexpression in cell invasion. We then performed Matrigel in vitro invasion assays with the overexpressing cell lines (Fig. 3a). A significant decrease in the invasion capacity was observed for both DU145 and LNCaP cell lines overexpressing nc886 relative to their corresponding controls (Fig. 3d).

### The expression of nc886 correlates with the expression of genes linked to tumor proliferation in PrCa

**Fig. 5:**
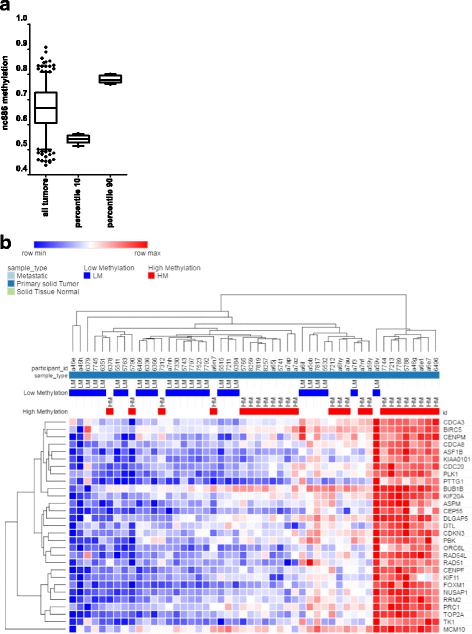
Association between TSS200 nc886 methylation status and the prostate cancer cell cycle progression (CCP) gene expression signature in the TCGA- PRAD cohort. **a** Box plot of the distribution of nc886 average promoter methylation in the total TCGA-PRAD dataset and the edge 10-percentile samples selected for the clusterization. **b** Heat map of the expression of the genes belonging to the prostate cancer cell cycle progression (CCP) gene expression signature [32] in 10th percentile low and 90th high nc886 promoter methylated samples of TCGA-PRAD. The heatmap was generated using the Euclidean algorithm clusterization with the Morpheus software

In order to study the molecular basis of the effect of nc886 in cell proliferation, we analyzed the putative association between the expression of nc886 and selected gene sets using the TCGA-PRAD cohort. Initially, we studied the expression of differentially expressed genes identified in a knock down of nc886 in esophageal, gastric and thyroid cell lines [18, 20, 21]. We did not find the predicted association between nc886 and these gene signatures in prostate samples (Additional file 4: Table S2). Indeed, none of the analyzed genes correlate with nc886 expression as described in the former reports and 18 out of 38 showed significantly negative correlation with TSS200 nc886 average methylation in the TCGA-PRAD cohort (*p*-value < 0.0001). In addition, based on our in vitro and in vivo phenotypic data, we looked at the expression of the genes belonging to the PrCa CCP proposed by Cuzick et al., a 31-gene subset of 126 previously identified cell-cycle-related genes [32]. The score derived from the CCP has been later shown to possess clinical value by several independent studies, and is commercialized as the Prolaris test (Myriad Genetics, Salt Lake City,Utah, USA). When we analyzed all the tumors of the TCGA-PRAD dataset, 6 out of 28 genes of the signature show a concordant significantly positive correlation with the TSS200 nc886 methylation (p-value < 0.0001) and none of them show a negative correlation with TSS 200 nc886 methylation (Additional file 5: Table S3). Additionally, we defined two group of samples (25 tumors each one) showing low and high nc886 promoter methylation (Fig. 5a); tumors at the 10th percentile (average beta-value 0.542 ± 0.003) were classified as low TSS200 methylation and consequently high nc886 expression, while tumors at the 90th percentile (average beta-value 0.780 ± 0.003) were classified as high TSS200 methylation and consequently low nc886 expression. As depicted in the Fig. 5b, low and high TSS200 nc886 methylation tumors tend to cluster based on the expression of the CCP signature. Furthermore, the transcripts belonging to the CCP signature showed increased expression in the high TSS200 methylation compared to the low TSS200 methylation group.

## Discussion

Nc886 has been recently shown to act as a tumor suppressor ncRNA in cholangiocarcinoma, esophageal carcinoma, gastric cancer and leukemia [15, 18, 20, 33]. The etiology of its downregulation in cancer has been linked to the methylation of its promoter in leukemia, colon, lung, gastric, bladder, breast and esophageal tumors [15, 16, 18–20]. Furthermore, nc886 has been proposed both as a tumor suppressor and as an oncogene, depending of the context and the tissue involved, as was recently showed in thyroid cancer [21]. Thus, a more comprehensive picture of nc886 action in cancer, including tissue specific differences and potentially specific molecular mechanisms is still required.

Here we present the first study of the role of nc886 in PrCa. We found an increased methylation of nc886 gene promoter in prostatic tumor tissue vs. its matched normal counterpart, analyzing the samples available at the TCGA-PRAD dataset and in the cohort of Kirby et al. A similar observation was made in leukemia, colon, lung, gastric, bladder, breast and esophageal tumors [15, 16, 18–20], supporting the hypothesis of increased nc886 promoter methylation as a recurrent event in the initiation step of solid tumors. Indeed, we found that the level of nc886 promoter methylation correlates with PrCa patient clinical evolution, reinforcing previous findings in gastric, lung, leukemia and esophageal cancer [15, 16, 18, 20]. Furthermore, the predominant medium and high nc886 methylation groups tissues, in both normal and tumor TCGA-PRAD samples, positively correlate with the clinical outcome of the disease. It is worth to note, that the so-called “normal adjacent” prostatic tissue from PrCa patients (Fig. 1a), may be in fact abnormally modified by tumor induced changes at the organ level [28], thus conclusions derived exclusively from this type of tissue should be taken with caution. Indeed, the study of Aryee et al. (Fig. 1b) reports lower levels of average nc886 promoter methylation in bona fide prostate normal tissue obtained from organ donors, suggesting that “normal tissue” adjacent to tumor tissue may had already undergone an increase in nc886 promoter methylation. Alternatively, methodological differences between the two studies may explain the divergence in the average methylation. The fact that another recent study using normal adjacent tissue reports nc886 promoter methylation comparable to TCGA-PRAD, favors the former interpretation. In addition, we found significantly lower levels of average nc886 promoter methylation in bona fide normal tissue obtained from organ donors in comparison with metastatic tissues. The finding of similar levels of TSS200 methylation in high methylated samples from normal and primary tumors in comparison with metastatic tissue, suggests that nc886 promoter methylation is a pre-requisite for tumor metastasis. Altogether, our results favor a tumor suppressor role for nc886 in several steps of PrCa tumorigenesis. It also indicates that nc886 silencing is a driver epi-mutation in PrCa. Finally, our findings point out to a potential use of nc886 for disease stratification in PrCa.

Our study also proves that the level of expression of nc886 in PrCa tissue is significant lower than in the normal counterpart. This goes in agreement with findings in other tissues, in which nc886 was proposed as a tumor suppressor gene [12, 15, 18, 20, 33]. In addition, we show that nc886 promoter methylation negatively regulates its transcript abundance in PrCa cell lines, as was shown previously in leukemia, gastric and esophageal tumors [15, 18, 20].

Although aberrant DNA hypermethylation in PrCa is a fundamental driver of tumor progression and overexpression of the DNMTs is a signature of disease origin and evolution, the mechanism responsible for the epigenetic silencing of nc886 in cancer has not been addressed so far. Among the three DNMTs, DNMT3B has been consistently shown to increase its levels in transformed vs. normal prostate tissue, both in patient tumors and in cell lines [34–37] and its expression increase along with adverse clinical parameters [36, 37]. Functional studies in siRNA cell lines, cadmium-transformed prostate epithelial cells and TRAMP mouse models [35, 38, 39], together with the association between PrCa risk and a polymorphism in DNMT3B leading to increased enzyme expression [40], have provided further support to this hypothesis. Thus, DNMT3B seems to be the most important DNMT driver in PrCa. Concordantly, we found a positive correlation between the fold change in expression of DNMT3B and nc886 promoter methylation in matched normal to tumor tissue in the TCGA-PRAD cohort, which favors DNMT3B involvement in nc886 promoter methylation during neoplastic transformation in the prostate. Nevertheless, further experiments in PrCa models are needed to prove the hypothesis.

Seeking the effect of nc886 deregulation in prostate tissue, we analyzed several PrCa cell lines finding support for the correlation between nc886 expression and methylation seen in the clinical samples. In addition, the cell lines show quite variable expression of nc886 in agreement with the patient data. Accordingly, an important heterogeneity in nc886 promoter methylation has been reported in several studies. The natural variation in the methylation of the locus in humans might be explained by imprinting and methylation in response to the peri-conceptional environment [19, 22, 23]. Nevertheless, the increase in the methylation of nc886 promoter in tumor vs. matched normal tissue regardless of the initial level of expression in the normal tissue, has strong support in the cancer literature [15, 16, 18–20]. In this context, we chose two cell lines with different levels of nc886 methylation and concomitant expression to cover the spectrum of nc886 variation in prostate tissue. In addition, these cells lines model the androgen sensitive (LNCaP) and androgen insensitive (DU145) forms of PrCa disease. We found that nc886 overexpression produces a significant decrease of the in vitro proliferation, possibly by a retention of the cells in the G2/M stage of the cell cycle. Additionally, when we assessed the effect of overexpression of nc886 in vivo we found a significant decrease in tumor growth. The decreased growth of nc886 overexpressing tumors was accompanied by a high percentage of necrosis, low number of mitosis and low tumor weight trends. Forced overexpression of nc886 causes a reduction in in vitro invasion through Matrigel. The concordant pattern of methylation and expression of nc886 observed together with the phenotype of the overexpressing cells strongly contributes to the idea that nc886 functions as a tumor suppressor gene in the prostate.

Then we looked for possible transcriptomic changes responsible for the proliferative effect of nc886 in PrCa. We observed that most of the expression of genes previously shown to be upregulated upon nc886 knock-down in cell lines models of esophageal, gastric and thyroid cancer, do not correlate with nc886 expression in prostate tumor tissues. This finding suggests that the molecular changes induced by nc886 deregulation in esophageal, gastric and thyroid cancer are different that those in the prostate, thus favoring a tissue specific model of nc886 action in cancer. Remarkably, we found that the CCP, a validated clinically useful PrCa proliferation gene signature, positively associates with nc886 expression in the TCGA-PRAD cohort. This goes in agreement with the negative effect of nc886 in cell proliferation observed both in vitro and in vivo using a gain-of-function of nc886 in PrCa cell lines and poses candidate genes that might be co-regulated with, responsive to or regulators of nc886. While different molecular mechanisms of nc886 action in cancer have been proposed, including the modulation of the PKR/NFkB pathway [12, 41] and the generation of microRNAs [16, 17], the relative contribution of nc886 and derived microRNAs in cancer has been hardly addressed in the literature. They could either modulate the same or completely independent cellular processes through diverse molecular pathways. Interestingly, some of the genes belonging to the CCP signature have mRNA motifs complementary to the seed region of the microRNAs potentially derived from nc886. Indeed, one of these has already been validated as a target gene in another tissue [16]. Although more work is necessary to elucidate the precise molecular mechanism of action of nc886 in the prostate cells, its association with the CCP at the level of gene expression in the clinical set reinforces its clinical relevance in PrCa disease.

## Conclusions

This investigation validates the presence of nc886 transcript in the prostate, and its downregulation during the progression of PrCa. The correlation between nc886 promoter methylation and several PrCa clinical parameters suggest its clinical importance in PrCa progression and is consistent with data previously reported for other cancers. The analysis of cell lines provided direct prove of nc886 regulation by DNA methylation and identifies DNMT3B as potentially responsible for the increase in nc886 methylation during malignant transformation in the prostate. Functional studies using a gain-of function of nc886 in PrCa cell lines exposed its negative influence on cell proliferation and invasion. The relevance of the proliferative effect in the clinical set is supported by the positive correlation between TSS200 nc886 methylation and the PrCa proliferation gene signature (CCP) in the TCGA-PRAD. These data point out to a tumor suppressor role nc886 in PrCa, whose expression is epigenetically silenced in cancer resulting in an increased cell proliferation and invasion through tissue specific molecular mechanisms.

## Additional files

Additional file 1: Figure S1. Hierarchical cluster based on nc886 methylation status of paired normal and tumor tissue of PrCa patients from TCGA-PRAD and Stanford datasets. Clusterization was performed by MEV [42] using Euclidean algorithm with default parameters. The beta-values of methylation of the 6 CpG sites comprise the TSS200 in the normal and tumor tissue considered as a unit. Each row corresponds to one patient whose identity is indicated at the right of the heatmap using the ID provided by the original study. Upper rulers indicate the amplitude of gene expression represented by the colors of the heatmap. (PDF 147 kb)

Additional file 2: Figure S2. Hierarchical cluster of 50 paired normal and tumor tissue of PrCa patients from TCGA-PRAD dataset. A. 50 normal prostate tissue B. 50 prostate tumor tissues. Clusterization was performed by Gene-E Euclidean algorithm using default parameters. The two major clusters, indicated as “high” and “medium” methylation, were selected for further clinical association studies shown in D and F. The average methylation of 10 CpG sites of nc886 promoter (including the 6 sites of the TSS200, 1 located at the gene body and 3 located 200-350pb upstream of the TSS200) is shown for normal (C) and tumor tissue (E). The clinical variables reported at the TCGA-PRAD were analyzed for correlations with the methylation status of nc886 promoter, and only the statistically significant associations found are shown. D. Average clinical T value is associated with the methylation status of the normal tissue (normal) of the patient. F. Average pathological T value is associated with the methylation status of the tumor tissue (tumor) of the patient. *P-value < 0.05; ** P-value < 0.01; *** P-value < 0.001 two-tailed t-test. (PDF 419 kb)

Additional file 3: Table S1. Correlation of the expression of the DNMTs with nc886 TSS200 methylation level in TCGA-PRAD tumor samples. (XLSX 9 kb)

Additional file 4: Table S2. Correlation of the differentially expressed genes identified in a knock down of nc886 in esophageal, gastric and thyroid cell lines and nc886 TSS200 methylation level in TCGA-PRAD tumor samples. (XLSX 11 kb)

Additional file 5: Table S3. Correlation of the expression of PrCa cell-cycle progression gene signature (CCP) and nc886 TSS200 methylation level in TCGA-PRAD tumor samples. (XLSX 10 kb)

## Abbreviations

CCP: Prostate Cancer Cell Cycle Progression Gene Signature
DMSO: Dimethyl Sulfoxide
GEO: Gene Expression Omnibus
H&E: Hematoxylin and Eosin
MTT: 3-(4,5-dimethylthiazol-2-yl)-2,5-diphenyl-2H-tetrazolium bromide
ncRNA: Non-Coding RNA
nt: Nucleotide
OD: Optical Density
PBS: Phosphate Buffer Solution
PRAD: Prostate Adenocarcinoma
PrCa: Prostate Cancer
pre-miR-886: Precursor of hsa-miR-886
qPCR: Quantitative Polymerase Chain Reaction
RT: Reverse transcription
TCGA: The Cancer Genome Atlas
TSS200: Region of 200 nt located upstream of the transcription start site
vtRNA: Vault RNA
