# Supplementary figures and images for: Nc886 is epigenetically repressed in prostate cancer and acts as a tumor suppressor through the inhibition of cell growth
Source: BMC Cancer. 2018 Feb 2;18:127. doi: 10.1186/s12885-018-4049-7 (PMC5797390; doi:10.1186/s12885-018-4049-7)

## TCGA

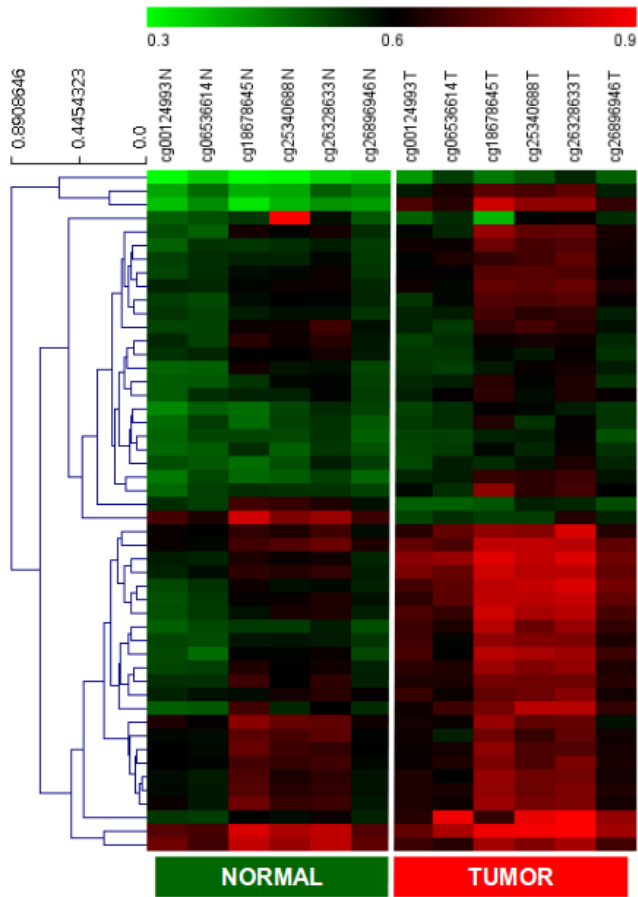

## STANFORD

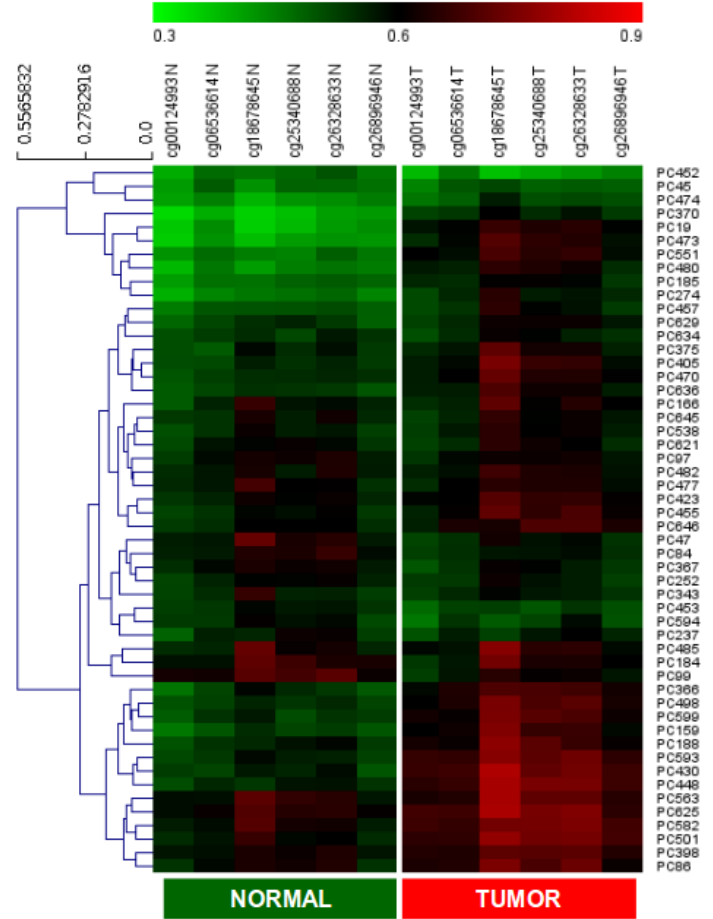

Supplement: Supplementary file 1 — Hierarchical cluster based on nc886 methylation status of paired normal and tumor tissue of PrCa patients from TCGA-PRAD and Stanford datasets. Clusterization was performed by MEV [42] using Euclidean algorithm with default parameters. The beta-values of methylation of the 6 CpG sites comprise the TSS200 in the normal and tumor tissue considered as a unit. Each row corresponds to one patient whose identity is indicated at the right of the heatmap using the ID provided by the original study. Upper rulers indicate the amplitude of gene expression represented by the colors of the heatmap. (PDF 147 kb) [file 12885_2018_4049_MOESM1_ESM.pdf]

**A**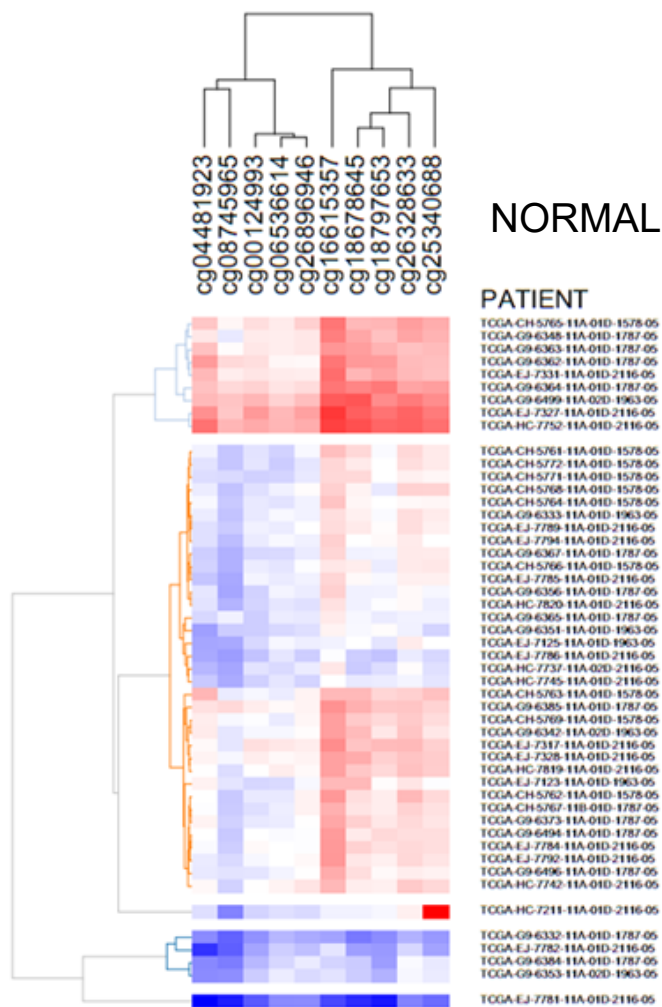**B**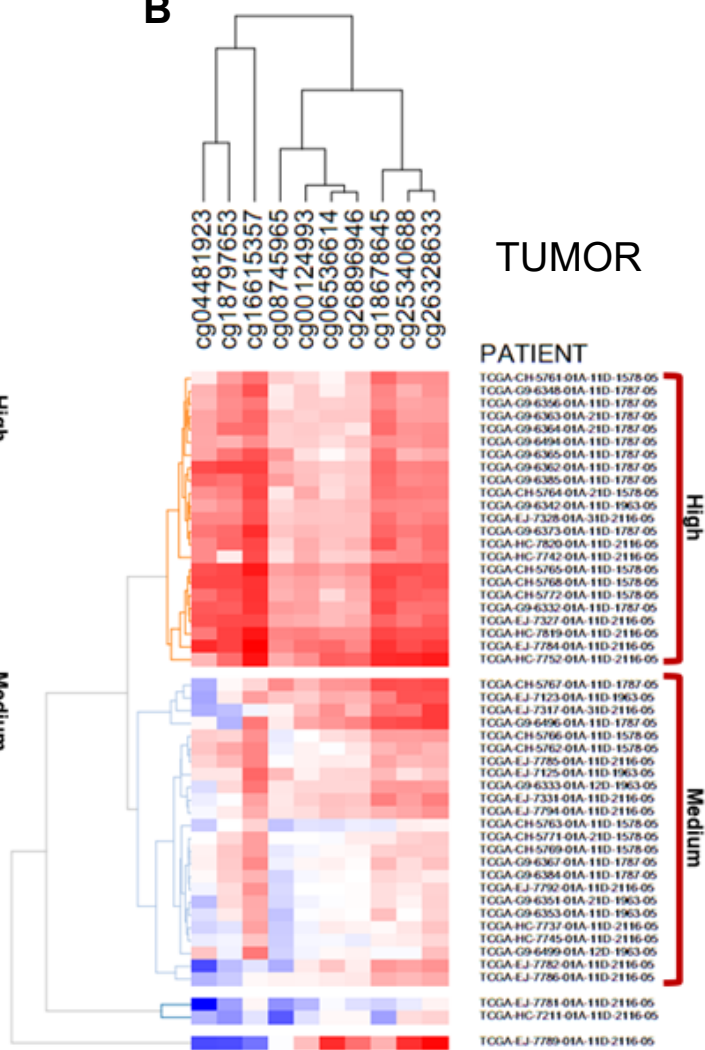**C**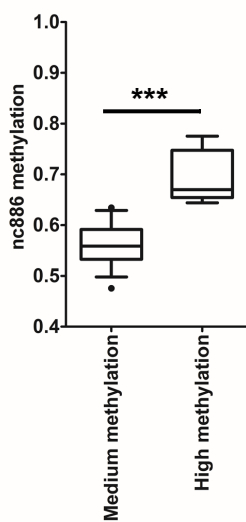**D**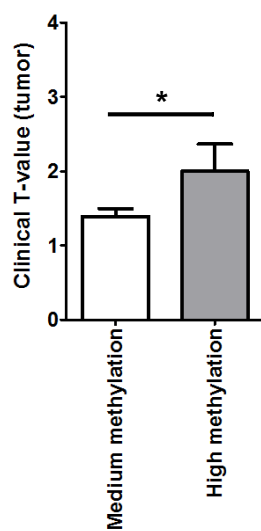**NORMAL****E**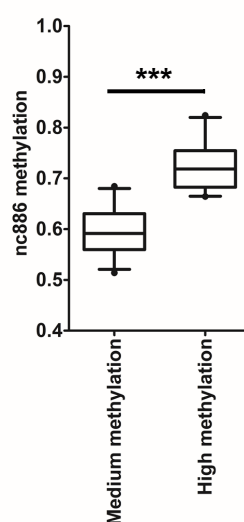**F**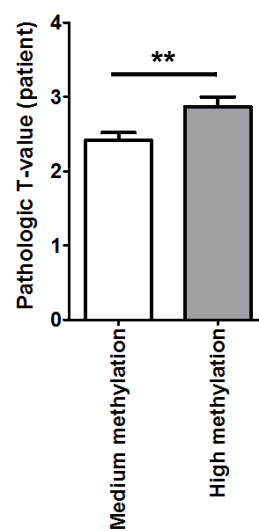**TUMOR**

Supplement: Supplementary file 2 — Hierarchical cluster of 50 paired normal and tumor tissue of PrCa patients from TCGA-PRAD dataset. A. 50 normal prostate tissue B. 50 prostate tumor tissues. Clusterization was performed by Gene-E Euclidean algorithm using default parameters. The two major clusters, indicated as “high” and “medium” methylation, were selected for further clinical association studies shown in D and F. The average methylation of 10 CpG sites of nc886 promoter (including the 6 sites of the TSS200, 1 located at the gene body and 3 located 200-350pb upstream of the TSS200) is shown for normal (C) and tumor tissue (E). The clinical variables reported at the TCGA-PRAD were analyzed for correlations with the methylation status of nc886 promoter, and only the statistically significant associations found are shown. D. Average clinical T value is associated with the methylation status of the normal tissue (normal) of the patient. F. Average pathological T value is associated with the methylation status of the tumor tissue (tumor) of the patient. *P-value < 0.05; ** P-value < 0.01; *** P-value < 0.001 two-tailed t-test. (PDF 419 kb) [file 12885_2018_4049_MOESM2_ESM.pdf]
